# Species distribution modeling to predict tsetse fly (*Glossina* spp.) habitat suitability in Kenya

**DOI:** 10.1186/s13071-025-06938-1

**Published:** 2025-09-24

**Authors:** Raphael Mongare, Stella Gachoki, Elhadi Adam, Emily Kimathi, Antoine M. G. Barreaux, Giuliano Cecchi, Seth Onyango, Nancy Ngari, Daniel Masiga, Elfatih M. Abdel-Rahman

**Affiliations:** 1 International Centre of Insect Physiology and Ecology (icipe), P.O. Box 30772, Nairobi, 00100 Kenya; 2 School of Geography, Archaeology and Environmental Studies, University of the Witwatersrand, Johannesburg, 2025 South Africa; 3 Intertryp, Université Montpellier, Centre de Coopération Internationale en Recherche Agronomique Pour Le Développement (CIRAD), Institut de Recherche Pour Le Développement (IRD), 34000 Montpellier, France; 4 Animal Production and Health Division, Food and Agriculture Organization of the United Nations, Rome, Italy; 5 Kenya Tsetse and Trypanosomiasis Eradication Council (KENTTEC), P.O. Box 66290, Nairobi, 00800 Kenya; 6 School of Agricultural, Earth, and Environmental Sciences, University of KwaZulu-Natal, Pietermaritzburg, 3209 South Africa

**Keywords:** Data science, Remote sensing technology, Machine learning, Vector-borne diseases, Animal, Health

## Abstract

**Background:**

African animal trypanosomosis (AAT) and human African trypanosomosis (HAT) are transmitted and spread primarily by tsetse flies (*Glossina* spp.) in sub-Saharan Africa. The animal disease poses significant challenges to agropastoral systems, including in Kenya, where 38 out of 47 counties are infested with eight species of *Glossina*. Climate change and human activities can also aggravate these infestations, putting rural-scale farmers who rely on agropastoral systems at a greater risk. Geographical gaps in existing entomological datasets limit a comprehensive understanding of tsetse fly distribution across the country, especially amid rapid landscape dynamics.

**Methods:**

This study aimed to predict the spatial distribution of tsetse flies habitat in Kenya using recent entomological data (i.e., tsetse fly occurrence records), satellite-derived environmental variables, landscape structure, demographic indicators, and species-distribution modeling techniques. We applied four machine learning (ML) algorithms—random forest (RF), support vector machines (SVM), maximum entropy (MaxEnt), and generalized linear models (GLM)—to predict tsetse flies habitat suitability. Additionally, we developed ensemble models that combine the predictive power of the four algorithms. Predictions were made at the genus level (*Glossina* spp.) and the species level for one priority species (*Glossina pallidipes*).

**Results:**

The models performed well with true skill statistic (TSS) and area under the curve (AUC) metric measures of 0.67 and 0.88 for *Glossina* spp. and 0.85 and 0.96 for *G. pallidipes*, respectively. The predictions indicated an estimated potential suitable area of about 26% of Kenya for *Glossina* spp. and 9% for *G. pallidipes*. Tsetse fly habitat suitability was positively correlated with increased sheep density, normalized difference vegetation index, and soil moisture. However, suitability declined when the maximum land surface temperature (LST) exceeded 40 °C and elevation increased above 400 m.

**Conclusions:**

These findings can help improve the targeting and, hence, the cost-effectiveness of surveillance and ultimately support an evidence-based progressive control of tsetse flies infestation in Kenya.

**Graphical Abstract:**

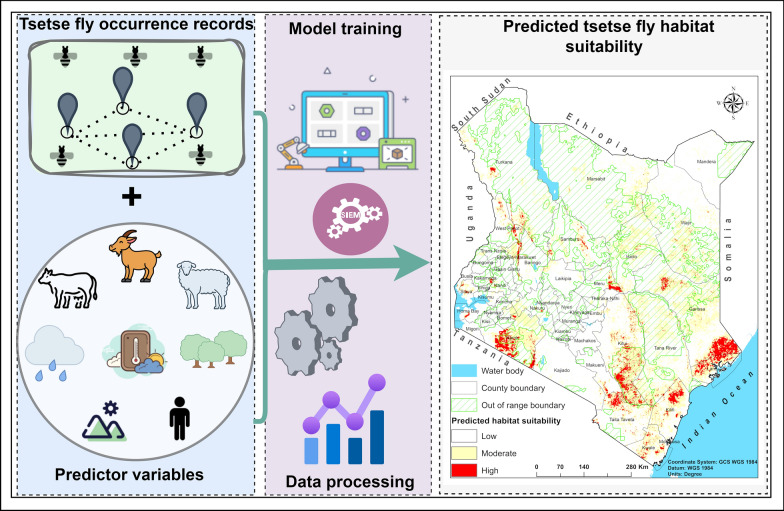

**Supplementary Information:**

The online version contains supplementary material available at 10.1186/s13071-025-06938-1.

## Background

Tsetse flies (*Glossina* spp.) transmit African animal trypanosomosis (AAT) and human African trypanosomosis (HAT), with profound impacts on animals and humans in the tropics [1, 2]. The disease-causing pathogens are transmitted cyclically through tsetse flies and can also be transmitted mechanically through biting flies (stomoxes and tabanids) from infected hosts to uninfected animals. The most common trypanosome species transmitted by tsetse flies to livestock include *Trypanosoma vivax*, *T. congolense*, *T. brucei* and *T. simiae*. By contrast, *T. evansi* and *T. vivax* have expanded their geographical range beyond the tsetse fly belt through mechanical transmission by other biting flies [3, 4]. Increased tsetse fly infestations have inhibited agricultural production, splitting the integration of crop-livestock systems and overwhelming economies and livelihoods among small-scale rural farmers relying on these production systems [5, 6].

In sub-Saharan Africa (SSA), at least 35 countries are presently considered to be infested by tsetse flies [7], with 10 economically important tsetse fly species spanning over two-thirds of the region. It is estimated that 309 million farmers rely on agricultural production systems of livestock to sustain their livelihoods in SSA. This threatens approximately 40% of total household income from livestock production, and about 70% of the farmers are highly exposed and vulnerable to the impacts of AAT and HAT [8]. In Kenya, it is estimated that over 130,000 km^2^ of land is infested with tsetse flies across 38 out of 47 counties, causing yearly economic losses of about US$ 0.20 billion [9]. The vast infestation comprises eight known tsetse fly species impacting tourism, wildlife, animal production, and human health, risking agrifood systems and rural economies that depend on livestock production [9]. Likewise, Kenya has several protected areas and conservancies across the landscape that provide immediate habitats for *Glossina* spp. Wildlife abundance in these areas offers a pool of reservoirs favorable for increased infection transmission rates [10].

Climatic and environmental factors influence the behavior, occurrence, and distribution of tsetse flies. For instance, temperature is a determining factor of tsetse fly survival and development. It influences feeding activity, reproduction rate, and mortality levels [11, 12]. Studies have shown that temperatures above 25 °C increase tsetse fly female mortality, while pupal development is affected mainly as temperatures increase or decrease beyond the extremes (< 16 °C and > 32 °C) [11, 12]. While precipitation does not directly correlate with tsetse fly distribution, it highly influences environmental factors that support tsetse fly development and survival, such as vegetation, humidity, and soil moisture. Well-established vegetation cover provides shelter and breeding sites for tsetse flies, modifies humidity and temperature levels, and regulates soil moisture, which is critical in pupal development [11]. However, excessive soil moisture and high amounts of rainfall can cause tsetse fly pupae mortality and wash away the buried ones through surface run-off in loose soils [11]. Moreover, areas experiencing variable climate shifts and human impact have shown increased disruptions of the optimal tsetse fly habitat conditions, reducing vector population and limiting its distribution.

Various methods have been developed and effectively used to manage tsetse fly and trypanosomosis [13, 14]. Notwithstanding, ecological alterations caused by increased landscape and climate dynamics and changes further hinder the effectiveness of tsetse fly management tools. This suggests that adopting evidence-based and locally tailored management measures would be optimal for enhancing and sustaining agricultural systems. For instance, Saini et al. [15] developed a tsetse fly repellent collar, an innovative and promising vector control technology that employs tactical mimicking of non-host odour profile to repel the flies. This technology has been tested among small livestock keepers in Kenya’s coastal region, demonstrating its potential for broader application [16]. Scaling up and expanding these control techniques is hindered by limited geographical gaps in understanding the tsetse fly distribution. Therefore, current and updated tsetse fly habitat distribution maps are essential for pinpointing ecologically suitable landscapes and prioritizing planning efforts for the national control of AAT.

Localized operations would continually suppress tsetse-related impacts and reduce the propagation rate of AAT among domesticated animals. In Kenya, highly localized tsetse fly and trypanosomosis distribution maps developed within a national atlas initiative presently support the deployment of control methods and novel technologies [9]. However, the atlas is affected by geographical gaps, and modeled maps could shed light on the possible vector and disease occurrence in areas not yet covered by field surveys. These modeled maps would provide potentially isolated tsetse fly habitat ranges amid the shifting climatic regimes and increased human activities. Integrating novel geospatial techniques with entomological data, ecologically relevant remote sensing variables, and predictive analysis through machine learning-based species distribution models (SDMs) offers a robust framework for identifying ecologically suitable habitats for the tsetse fly. Many studies have revolved around the understanding of tsetse fly control mechanisms [15], trypanosome infection rates, prevalence, and transmission risks [17], and tsetse fly distribution at distinct stages in hotspot areas [18]. Nevertheless, a few studies have explored and documented the national spatial distribution of tsetse flies, calling for the development of spatial distribution maps at a landscape scale [9, 18, 58]. This will update the long-relied-upon knowledge that has informed planning for nearly two decades, enhancing our understanding of tsetse fly habitat dynamics in a rapidly changing environment.

Predictive modeling techniques establish relationships between species occurrence and biophysical and ecological conditions, enabling the identification of suitable landscapes shaped by complex, interacting processes that influence species abundance and distribution. Species distribution machine-learning predictive approaches have been applied in numerous studies to evaluate arthropod habitat suitability utilizing satellite-based predictors over space and time [18, 19]. Individual SDM approaches have been successfully implemented in predicting the distribution of insects at a landscape scale [20, 21]. In other words, the applied state-of-the-art machine learning and species modeling algorithms help handle complex nonlinear ecological relationships to understand ecologically viable areas for species distribution [22]. Several of the widely used SDM techniques include support vector machines (SVM) [23], generalized linear models (GLM) [24], maximum entropy (MaxEnt) [25], random forest (RF) [26], and genetic algorithm [27].

The use of individual SDM algorithms to predict and model species distribution has been increasingly associated with uncertainties in model outputs [28, 29]. The individual models present variations in predictions compared with the ensemble of multiple algorithms, which is reported to have powerful predictive performance [29, 30]. This approach utilizes the highest predictive ability from each algorithm to compute a weighted outcome that counters and minimizes generalization and variations from single algorithm predictions [28]. It is envisioned that such outcomes are more robust, considering the novel integration of individual algorithms’ predictive ability and strength to generate more reliable and accurate results [28]. However, the combination of multiple SDM algorithms should be implemented cautiously, as it is not necessarily apparent that combining several algorithms yields better predictions. Therefore, this study aimed to predict the habitats of tsetse flies (*Glossina* spp. and *G. pallidipes*) in Kenya using an ensemble SDM approach. We constructed ensemble models combining four algorithms to mitigate prediction weaknesses in individual algorithms and provide reliable predicted habitats of tsetse flies in Kenya.

## Methods

### Study area

**Fig. 1:**
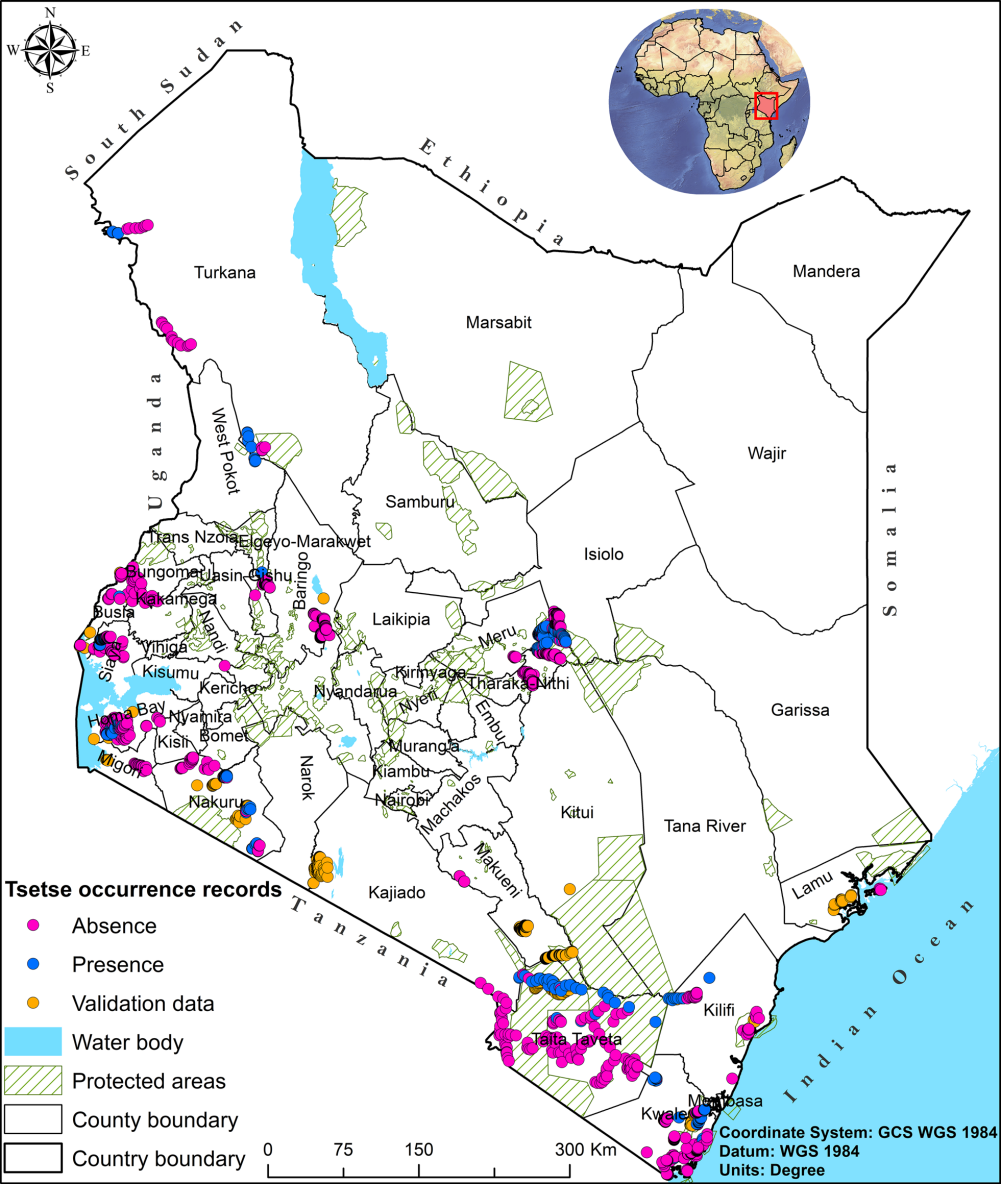
Location of Kenya as the study area showing the distribution of tsetse flies occurrence records across the landscape (colored dots), distribution of protected areas (hatched green areas), and neighboring countries in the East Africa region. Tsetse occurrence records were sourced from the Kenya Tsetse and Trypanosomiasis Eradication Council (KENTTEC) [9] and the International Centre of Insect Physiology and Ecology (icipe) repositories. Occurrence records collected between 2017 and 2020 (i.e., points shaded pink and blue) were used to build the models, and records collected between 2021 and 2023 (i.e., points shaded orange) were used to validate the model outputs

The study area covered the whole of Kenya, which lies on longitudes 33° 55′ E and 41° 55′ E and latitudes 04° 45′ S and 05° 25′ N, spanning an estimated area of 582,646 km^2^ that is characterized by forests, savannah, woodlands, grasslands, wetlands, and water bodies (Fig. 1). These land cover types provide suitable habitats for various tsetse fly species [18]. In Kenya, protected areas cover an estimated 8% of the total land area, comprising 26 national parks and 30 national reserves that span different climatic zones [31]. Wildlife species such as elephants, buffaloes, lions, leopards, zebras, giraffes, rhinos, and impalas dominate these wildlife conservation areas, offering a variety of alternative blood meal sources for tsetse flies. Further, approximately 80% of Kenya’s land is classified as semi-arid and arid, resulting in an overreliance on agricultural production systems to feed and sustain a population of more than 47 million people [32, 33]. The predominant agricultural production systems include pastoralism, ranching, feedlots, and agropastoralism, which are widely practiced across communal lands used as grazing areas by many communities. In addition, the country experiences two rainfall seasons: the long rainy season from March to May and the short rainy season from October to December. The rest of the year is cool and dry. Rainfall distribution varies significantly across regions, averaging between 250 and 2500 mm, and temperature ranges from 10 to 30 °C [34].

### Method overview

**Fig. 2:**
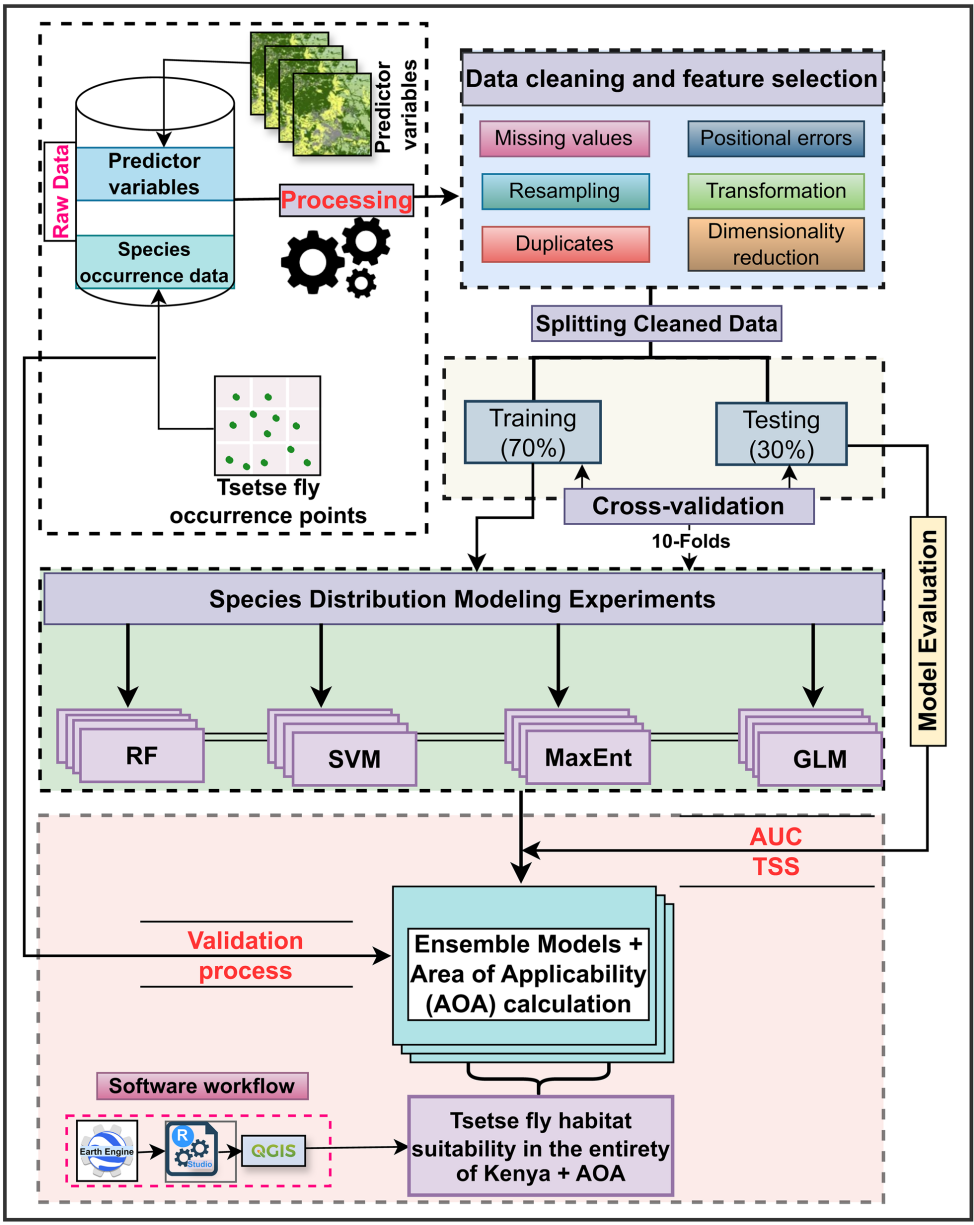
A flowchart showing a methodological approach applied in modeling Kenya’s potential tsetse flies habitat distribution. A total of four modeling algorithms employed include random forest (RF), support vector machines (SVM), maximum entropy (MaxEnt), and generalized linear models (GLM). *AUC* area under the curve, *TSS* true skill statistic, *AOA* area of applicability, *QGIS* Quantum Geographic Information System

The overall methodological approach of this study is described in Fig. 2. Species-level tsetse fly occurrence data accessed from the Kenya Tsetse and Trypanosomiasis Eradication Council (KENTTEC) and the International Center of Insect Physiology and Ecology (icipe) were used to predict the habitat suitability for tsetse flies. Predictor variables relating to environmental, topographic, demographic, animal, and landscape structure were extracted from readily available sources as monthly composites aggregated annually (*n* = 118) from 2017 to 2020. Variance inflation factor (VIF) analysis was employed to mitigate redundancy and multicollinearity among the predictor variables. Only variables with VIF values less than 10 were retained for further analysis. The sdm package [28] in the R programming environment [35] was utilized to train and fit the four models individually.

The ensemble models were constructed by integrating models that achieved an area under the curve (AUC) > 0.80 from the four individual fitted models. These ensemble models were used to develop national tsetse fly habitat suitability maps in Kenya. In this case, individual models were weighted on the basis of their performance (i.e., AUC score), and models with high performance had a higher influence on the final predictions. While the model’s performance was prioritized, model diversity was also considered to enhance the robustness of the ensemble. Models’ accuracy was evaluated using the AUC and true skill statistic (TSS) for the trained models. To further assess the models’ performance, an independent dataset was used to compute the confusion matrix and F1 score. Since the spatial prediction areas extended beyond the sampled points, the ensemble.novel function from the BiodiversityR package [36] was used to identify novel areas where environmental conditions fell outside the range of the training data, thereby limiting prediction reliability in those zones.

### Tsetse fly occurrence records

The main source of tsetse fly occurrence records for this study was the national atlas of tsetse flies and AAT for Kenya [9]. The atlas is developed by KENTTEC, the institution responsible for monitoring tsetse flies and controlling the associated diseases, covering all the main tsetse fly belts in Kenya. The atlas data collected between 2017 and 2020 were used for model training and testing, while data collected between 2021 and 2023 were used as an independent dataset for model output validation. Further detailed information on the tsetse fly occurrence data can be found in Ngari et al. [9]. Supplementary data from the Shimba Hills National Reserve, a critical tsetse fly hotspot in Kenya’s coastal region, were provided by icipe. Overall, a total of 2038 presence-absence records covering known tsetse fly belts across the country were provided. These records included the six most common tsetse fly species in Kenya (i.e., *Glossina pallidipes*, *G. brevipalpis*, *G. swynnertoni*, *G. longipennis*, *G. fuscipes*, and *G. austeni*) [9].

Data were cleaned to remove duplicates, check for missing values in the environmental predictor variables, and identify misaligned coordinates. After cleaning, 1,558 records were retained for the 2017–2020 dataset. These records were split into two subsets: training and testing, where 70% (*n* = 1,090) was used to train the model, and 30% (*n* = 468) was used as test data to evaluate model performance. For the 2021–2023 dataset, we retained 1,583 records after cleaning. The records were categorized into two data sets of genus-level models, herein as *Glossina* spp. (i.e., all tsetse fly species records) and a species-level model for *G. pallidipes* to predict suitable tsetse fly habitat distribution nationally. The genus-level model leveraged limited data from each species to generate a landscape-level outlook for *Glossina* spp., while the species-level model predicted habitat distribution for a single species (*G. pallidipes*). This species is widely reported as a dominant species in Kenya [37]. Tsetse fly occurrence records were used as the response variable in the model development process.

### Predictor variables acquisition and processing

Various environmental, topographic, demographic, animal, and landscape factors have been previously associated with providing suitable conditions for tsetse fly occurrence [18]. These factors and/or their surrogates are freely available from various providers at a medium-to-coarse resolution. These variables can be obtained to represent and infer the biophysical conditions of vector occurrence. Satellite-based environmental, topographic, demographic, animal, and landscape datasets were obtained from various sources at different resolutions, as summarized in Additional file 1: Table S1. These variables include normalized difference vegetation index (NDVI), land surface temperature (LST), air temperature, precipitation, soil moisture, surface soil moisture, subsurface soil moisture, soil temperature, skin temperature, surface run-off, sand content, elevation, human population density, cattle density, sheep density, goat density, distance to protected areas, and topographic wetness index. The selection and consideration of predictor variables were determined on the basis of their relevance to tsetse fly ecology and behavior through expert knowledge and intensive literature review. Environmental variables were sourced as minimum, median, maximum, and sum, matching the data collection period, as summarized in Additional file 1: Table S1.

These variables were subset to the whole of Kenya (our modeling area), scaled, projected, and resampled to 1 km spatial resolution using the resample function from the raster package [38] in the R programming environment [35]. Using tsetse fly occurrence data, an amalgamated database was created, linking each observation to the predictors at that site. This database was used in the subsequent analysis, which included multicollinearity assessment, model fitting, and testing.

### Multicollinearity test and variable selection

Environmental and climatic variables are usually correlated, exhibiting high redundancy, increasing uncertainty, and affecting the predictive performance of SDMs. Statistically significant predictors with less correlation are preferred in predictive analysis to make it easy to determine the realized relationship between predictor and response variables [39]. This allows for better generalization and reduces the possibility of model overfitting. We performed a multicollinearity analysis using VIF to measure the degree of correlation [40]. Predictor variables with a VIF value of less than 10 were retained, considering their relevance to influence tsetse fly occurrence. Variables with a VIF value greater than 10 are considered linearly correlated and can be problematic in predictive analysis [41]. The retained predictor variables were evaluated further statistically in a stepwise manner using the stats package in R programming to retain significant variables based on the least Akaike information criterion (AIC) value [35].

### Species distribution modeling

In this study, four SDM models were trained to compare their predictive power in predicting tsetse fly habitat suitability in Kenya. These algorithms include RF, SVM, MaxEnt, and GLM (Table 1). The algorithms were selected on the basis of their applicability and flexibility to model complex ecological interactions with excellent performance. The sdm package, which presents a single, extensible, and reproducible framework constituting SDM algorithms (*n* = 15), was used to train the models [28] in the R programming environment [35]. The package supports combining all or a selected number of algorithms to perform and compare different modeling algorithms’ diversity relating to species distribution and biophysical conditions [28].

**Table 1:** Species distribution models implemented in this study, as well as their short descriptions

Models	Short description
Random forest (RF)	RF is a machine learning ensemble algorithm that combines many classification trees, each of which fits into a bootstrapped dataset sampled randomly to obtain a majority vote of the popular class [26]. It is robust in forecasting species populations using abundance data [46] and handles presence/absence datasets in ecology as well. RF handles nonlinear parameters and is less prone to data dimensionality [26]
Support vector machines (SVM)	SVM is a machine learning algorithm with a high generalization ability of features to obtain optimal decisions through marginal maximization of complex datasets. It can handle linear and nonlinear interactions between the response and predictor variables [47]
Maximum entropy (MaxEnt)	MaxEnt is a presence-only machine learning model that predicts the probability of geographical suitability by finding a distribution widely spread out or nearly uniform within a set of environmental constraints. It can handle categorical and continuous environmental parameters [25]
Generalized linear models (GLM)	GLM is a regression model that can handle nonlinear data structures [24]. This approach uses a link function to fit the presence/absence of ecological data, and its flexibility allows it to be applied in ecological studies [48]

In this case, variables retained after the multicollinearity analysis were used to develop tsetse fly habitat suitability models at two levels (i.e., genus level: *Glossina* spp. and species level: *G. pallidipes*). The ensemble function within the sdm package was used to fit the resulting predictive models (i.e., for RF, SVM, MaxEnt, and GLM algorithms) for both genus and species modeling scenarios. To generate a robust ensemble model, a weighted average approach based on the AUC metric was applied, assigning respective AUC scores as weights to each model replicate (i.e., ten replicates per algorithm) based on a tenfold cross-validation method [28, 42]. Only model replicates with AUC values of 0.80 or higher were included in the ensemble weighting process [30]. All 40 model replicates (10 per each of the four algorithms) met the threshold of AUC ≥ 0.80. Then, we averaged the 40 AUC-weighted model replicates to generate a robust ensemble model. This weighted average method has been suggested to develop high-performing ensembles with improved predictive accuracy [28]. We used an AUC-weighted ensemble model because AUC tends to be an appropriate metric when comparing multiple models across species or areas with an unbalanced dataset [43], which is the case in this study. A TSS-weighted ensemble model, therefore, was not performed in this study. For consistency, the same approach was applied to produce ensemble models predicting the potential spatial distribution models of tsetse fly habitats for *Glossina* spp. and *G. pallidipes* in Kenya. Variable relative importance in predicting tsetse fly habitat was established using the getVarImp function in the sdm package, which relies on two metrics, i.e., correlation coefficient (R) and AUC metrics [28]. The rcurve function generated dependence plots, which highlighted the effect of each variable on the tsetse fly occurrence.

To generate potential spatial habitat maps, the threshold optimization criterion, i.e., maximum (sensitivity + specificity), was used to classify the predictions into suitable and unsuitable ranges [44]. The suitable ranges were then classified into three categories (i.e., low, moderate, and high) based on the natural breaks (Jenks) as described in [45]. The method resulted in natural breaks of < 0.32, 0.32–0.55, and > 0.55 for low, moderate, and high tsetse fly habitat suitability classes, respectively. For prediction reliability, we computed novel conditions (zones) to identify areas with limited applicability, given the training data as a reference. The ensemble.novel function from the BiodiversityR package was used to delineate areas where predictions apply across the entire country on the basis of environmental conditions used to train the models [36].

### Model performance evaluation and validation assessment

We used a tenfold cross-validation approach within the sdm package to evaluate the models’ performance, in which the dataset was split into 70% for training and 30% for testing. The accuracy metrics from the ten model replicates for each algorithm were averaged to obtain an overall performance estimate. We evaluated the accuracy of our models using the AUC metric. Additionally, we computed the TSS values to get a complementary insight into the models’ performance and reliability. The AUC values range from zero to one, where scores close to zero demonstrate poor prediction, and those close to one indicate excellent predictions. Typically, AUC values greater than 0.50 demonstrate better model prediction performance. In addition, we used TSS, which accounts for commission and omission errors through a combination of specificity and sensitivity. TSS values range from −1 to +1, where values smaller than or equal to zero indicate a poor agreement with observations, and values close to +1 indicate a perfect agreement between the observed and predicted values [49]. Further, the independent presence–absence records collected from 2021 to 2023 (*n* = 1,583) by KENTTEC were used to validate the performance of the models at another point in time. The records were used to extract predicted suitability scores for tsetse flies to compute a confusion matrix, elaborated in Table 2, where *a* and *d* are correct predictions, and by contrast, *c* and *b* are regarded as prediction errors [50]. Moreover, we computed the F1 score, the harmonic mean of precision and recall, to evaluate the predictive performance of the ensemble models using an independent dataset, as shown in Eq. 1. The F1 score utilizes confusion matrix elements to compute the precision and recall metrics of the model’s performance.

1 $${\text{F1 score = 2}}\, \cdot \,\left( {\frac{{\text{Precision * Recall}}}{{\text{Precision + Recall}}}} \right)$$

**Table 2:** The confusion error matrix elements^a^ are used to evaluate the predictive ability of species distribution models

Predicted	Actual	
	Positive	Negative
Positive	*a*	*b*
Negative	*c*	*d*

^a^Elements:

*a* = locations where the species is present and predicted to be present

*b* = locations where the species is absent but predicted to be present

*c* = locations where the species is present but predicted to be absent

*d* = locations where the species is absent and predicted to be absent

## Results

### Model performance assessment

**Fig. 3:**
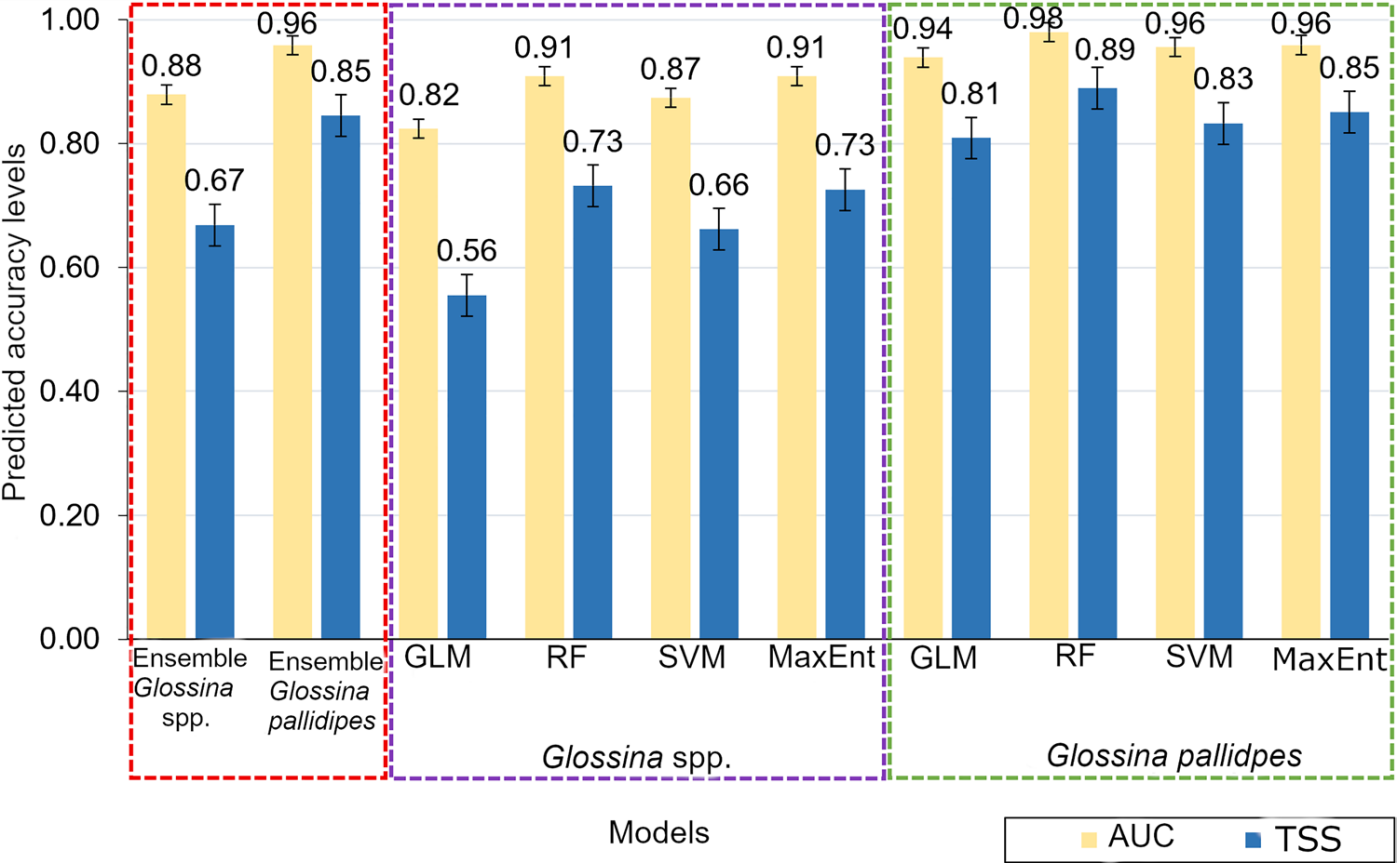
Accuracy metrics of the generalized linear models (GLM), random forest (RF), support vector machines (SVM), maximum entropy (MaxEnt), and ensemble models based on true skill statistic (TSS) and area under the curve (AUC) metrics. The three dotted lines in red, purple, and green indicate the various models, namely ensembles (i.e., *Glossina* spp. and *G. pallidipes*) and individual *Glossina* spp. and *G. pallidipes*, respectively

The RF and MaxEnt models showed similar and the highest AUC and TSS for predicting the *Glossina* spp. habitat suitability, while for *G. pallidipes*, RF had the highest accuracies (Fig. 3). RF had the highest performance and GLM performed the least, but with considerable performance across the modeling scenarios (Fig. 3). We combined individual models’ predictive outcomes for *Glossina* spp. and *G. pallidipes* to produce ensemble models. The ensembles achieved high accuracies, indicating an accurate prediction of *Glossina* spp. and *G. pallidipes* habitat distribution (Fig. 3). Evaluation of ensemble predictions with an independent dataset showed better performance using a confusion matrix analysis (Table 3) and F1 scores of 0.75 and 0.63 for *Glossina* spp. and *G. pallidipes*, respectively.

**Table 3:** A confusion error matrix for evaluating the predictive accuracy of *Glossina* spp. and *G. pallidipes* models using an independent dataset of presence-absence records collected from 2021 to 2023

	Accuracy (%)	
	Presence	Absence
*Glossina* spp.		
Suitable	78.5	28.7
Unsuitable	21.5	71.3
*G. pallidipes*		
Suitable	61.9	19.5
Unsuitable	38.1	80.5

### Variable selection, importance and association

**Fig. 4:**
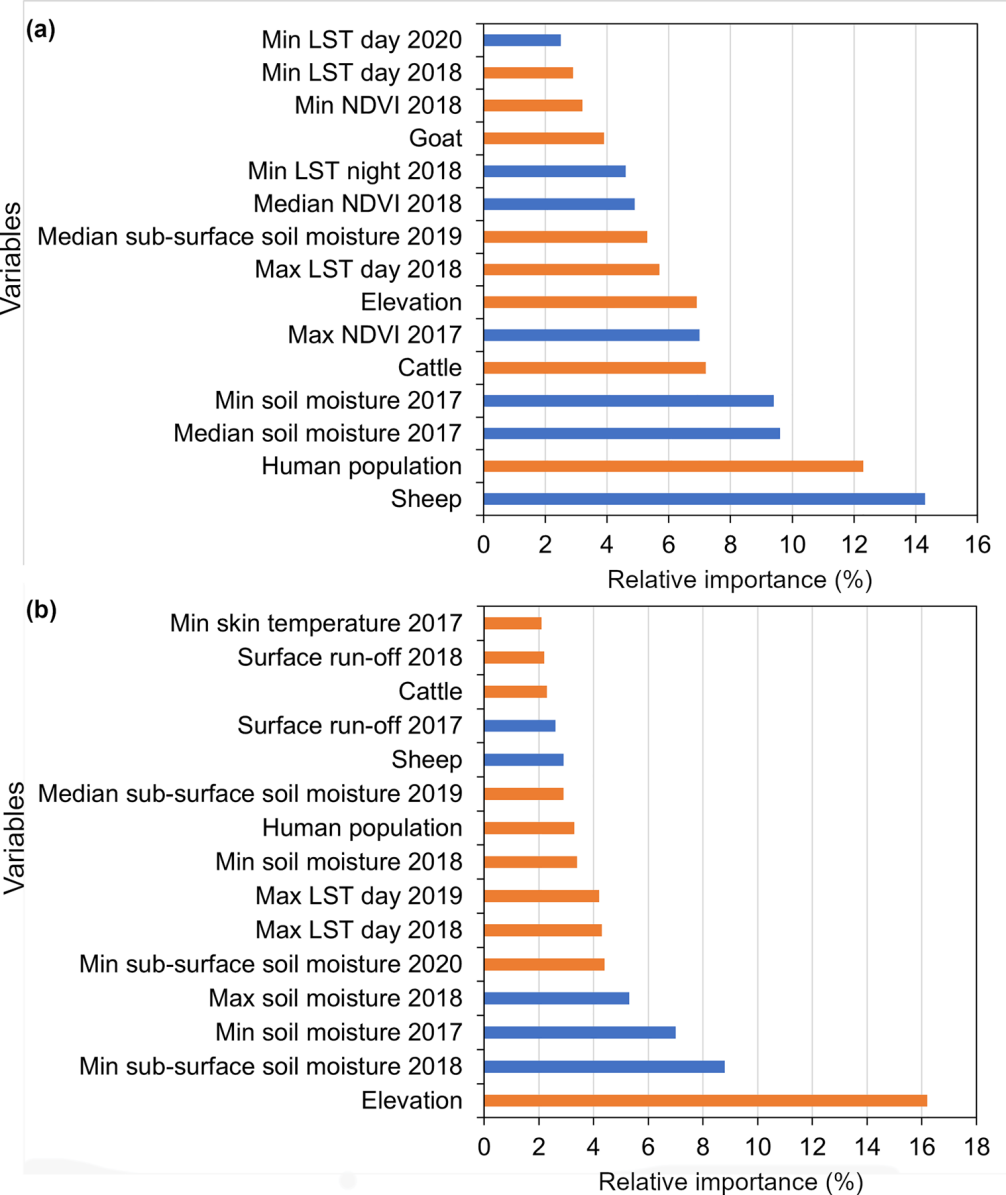
The most relevant variables and their association in predicting habitat suitability for (**a**) *Glossina* spp. and (**b**) *G. pallidipes* ensemble projections in the whole of Kenya. The blue bars indicate variables with a positive association, while the orange bars indicate a negative association with tsetse flies distribution. *min* minimum, *max* maximum, *med* median, *LST* land surface temperature, *NDVI* normalized difference vegetation index

**Fig. 5:**
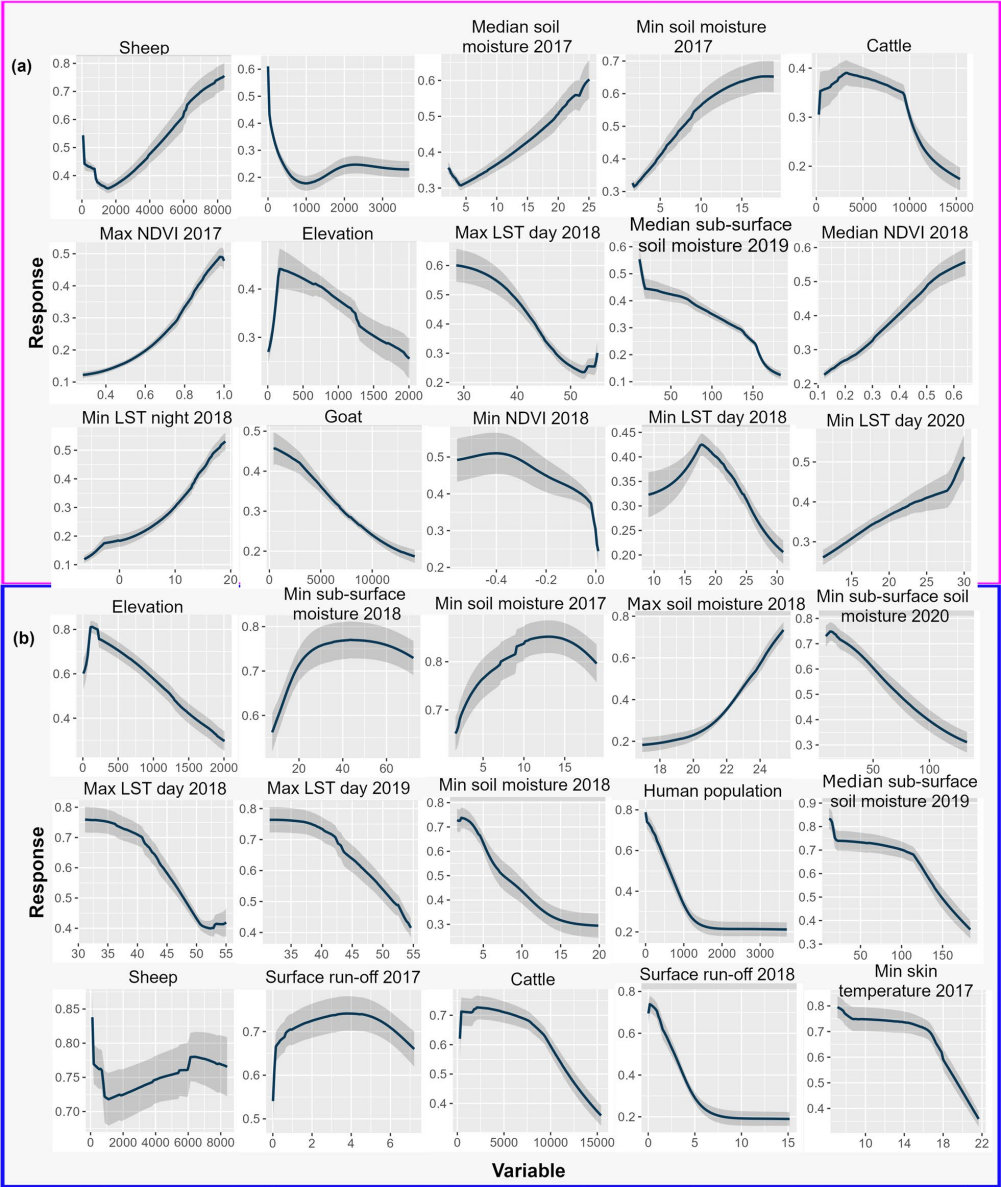
Response curves showing the interaction between predictor variables and tsetse flies occurrence in predicting habitat suitability for (**a)** *Glossina* spp. and (**b)** *G. pallidipes* ensemble projections in the whole of Kenya. *min* minimum, *max* maximum, *med* median, *LST* land surface temperature, *NDVI* normalized difference vegetation index

A total of 24 variables were retained following VIF and stepwise regression analyses (Additional file 2: Fig. S1). Variations were observed in variable importance and relationship to tsetse flies occurrence across experiments depending on the modeling technique and level (genus/species) of prediction, as shown in Fig. 4 and Additional file 3: Fig. S2. In the genus-level predictions, sheep and human population densities, soil moisture, cattle, and NDVI are the five most important variables (Fig. 4 and Additional file 3: Fig. S2). In the species-level predictions, elevation, soil moisture, LST, and human population densities were the four most important variables (Fig. 4 and Additional file 3: Fig. S2). Regardless of the genus or species, tsetse fly habitat suitability is positively associated with sheep density of 1,000–2,000 km^−2^. By contrast, tsetse flies habitat suitability showed a negative relationship with cattle and human population densities of more than 7,500–10,000 and 1,000 km^−2^, respectively (Fig. 5). However, topographic, edaphic, vegetation, and temperature variables showed mixed associations (positive and negative) with tsetse fly habitat suitability. Elevation less than 400 m, minimum and median soil moisture between 5 and 25 mm, and maximum and median NDVI above 0.6 favored tsetse fly habitat suitability, while subsurface soil moisture exceeding 40 mm reduced tsetse fly habitat suitability.

### Tsetse fly spatial habitat distribution

**Fig. 6:**
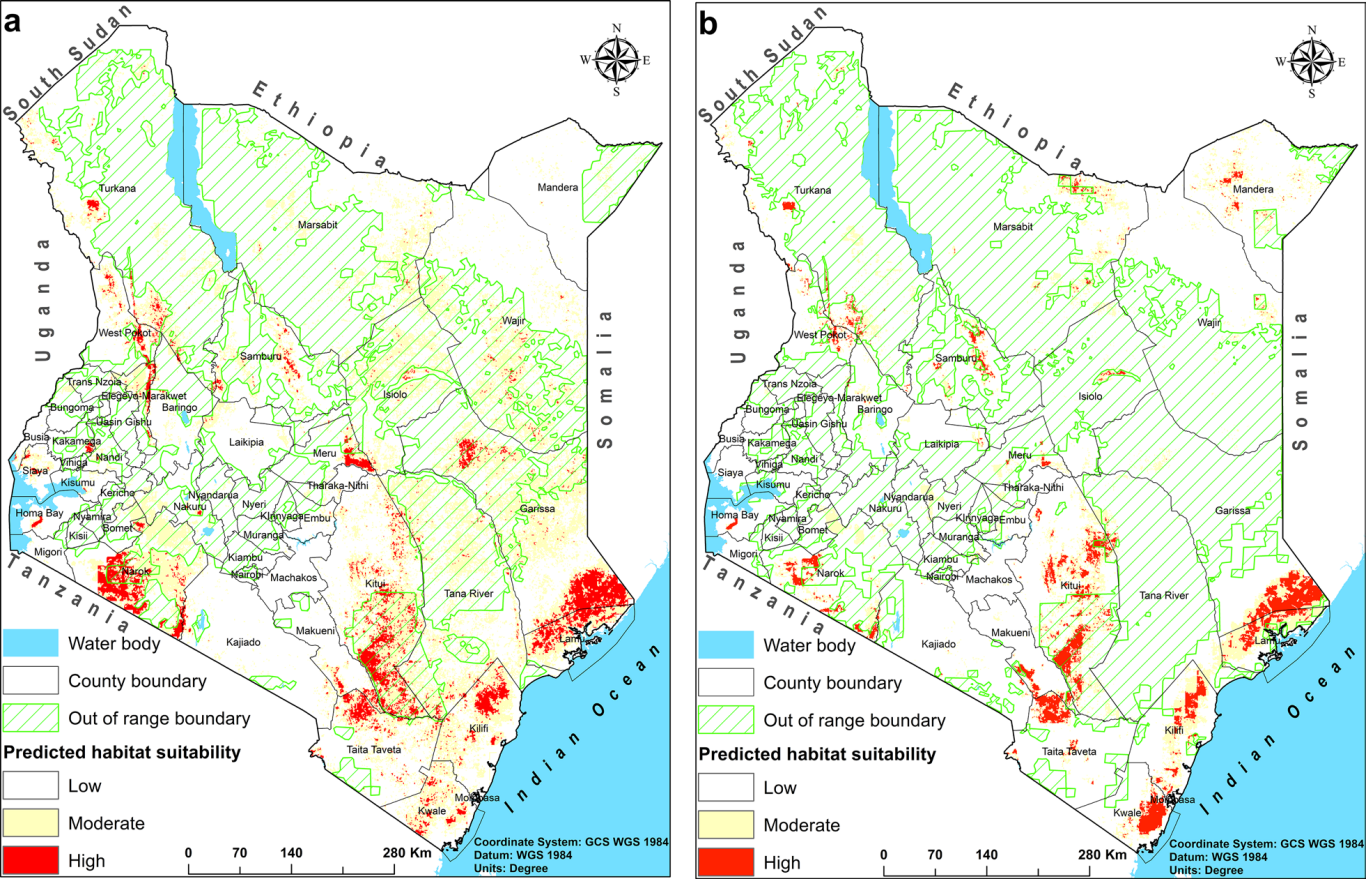
Tsetse fly spatial habitat suitability in the whole of Kenya was predicted using ensemble models where (**a)** *Glossina* spp. and (**b)** *G. pallidipes*. The ensembles combined the predictive power of four species distribution modeling algorithms, including random forest (RF), support vector machines (SVM), and maximum entropy (MaxEnt) and generalized linear models (GLM). The white shade indicates a low habitat suitability (< 0.32), the yellow shade indicates a moderate habitat suitability (0.32–0.55), and the red shade indicates a high habitat suitability (> 0.55). The green grids show novel zones—areas outside the range of environmental conditions used in model training

The predicted area coverage for *Glossina* spp. and *G. pallidipes* suitable habitat is estimated at 26% and 9% of the Kenya landscape, respectively (Table 4). The four SDMs demonstrated varied predictions across modeling experiments (Additional file 4: Fig. S3). The SVM, MaxEnt, and GLM algorithms indicated high levels of prediction, capturing known hotspots with notable variations. Visually, the RF demonstrated over- and under-prediction, although known tsetse fly hotspots were accurately predicted. We combined all four algorithms to construct ensemble models of *Glossina* spp. and *G. pallidipes* based on their overall performance. The ensemble models combined and estimated superlative predictive outcomes of the four models to predict tsetse fly habitat suitability (Fig. 6). The predictions indicated that *Glossina* spp. habitat is extensively distributed in the southern parts of Kenya, with reduced suitability for the northern parts. Moderate (0.32–0.55) to high (> 0.55) suitability is predicted in the eastern and more extensively towards the coastal regions, i.e., Kwale, Kilifi, Lamu, Garissa, Taita Taveta, and Kitui counties. Narok, Homabay, Kajiado, Busia, Siaya, Kakamega, Nakuru, Bomet, West Pokot, and Elgeyo-Marakwet counties in the Rift Valley and western regions demonstrated moderate-to-highly fragmented tsetse fly habitat suitability.

**Table 4:** The relative predicted distribution range for *Glossina* spp. and *G. pallidipes* (km^2^) and the total percentage of their suitable habitats in the whole of Kenya

Tsetse fly	Predicted suitable habitat (km^2^)	Landscape coverage (%)
Genus (*Glossina* spp.)	152,722	26.9
Species (*G. pallidipes*)	52,700	9.3

By contrast, the central region counties of Meru, Tharaka Nithi, Laikipia, and Embu exhibited patches of high habitat suitability for tsetse fly occurrence. Turkana county demonstrated relatively larger portions of highly suitable patchy areas compared with Marsabit, Samburu, and Wajir, which had moderate tsetse flies habitat suitability with small, fragmented patches. The *Glossina* spp. habitat is predicted to be high inside and around protected areas, community forest reserves, conservancies, rangelands, and segregated highlands in Kenya (Fig. 6a). National parks such as Maasai Mara, Tsavo East and West, Ruma, and Meru are identified as highly potential habitats for tsetse flies. High habitat suitability was also predicted in national reserves such as Shimba Hills, Dandara, Boni, Arubuko Sokoke, Nguruman escarpments, and Kibwezi forest, especially along the coastal belt.

Similarly, the distribution of suitable habitats for *G. pallidipes* in Kenya is fragmented, primarily concentrated around wildlife-protected areas. Unlike the broader habitat distribution of other *Glossina* spp., suitable habitats for *G. pallidipes* are limited to smaller, highly suitable areas. These areas include Tsavo East and West National Parks, the coastal counties of Kilifi, Kwale, and Lamu, Maasai Mara National Park in Narok county, Nguruman on the Kajiado-Narok border, Ruma National Park in Siaya County, Meru National Park in Meru county, and Turkana, Marsabit, Mandera, and Samburu counties, as well as surrounding conservancies in Laikipia and Isiolo counties (Fig. 6b).

Habitat distribution exhibits variations between low and moderate altitudes across the landscape with high-altitude areas, including the central region, except for several regions in Embu, Meru, and Laikipia, which are generally unsuitable (Fig. 6). These areas experience relatively low temperatures throughout the year. As a result, suitable habitats are concentrated in the east to southeast, with fragmented suitability in the west, southwest, and several regions in central Kenya. Habitat distribution in the country’s northern parts is sparsely distributed, with suitable areas moderately to highly segregated in Turkana, Marsabit, Samburu, Wajir, and West Pokot counties. The area of applicability analysis indicated that predicted suitability in the counties of Garissa, Wajir, Kitui, and parts of Narok remains uncertain with respect to tsetse fly occurrence (Fig. 6a, b).

## Discussion

This study aimed to develop spatial predictive maps to assess tsetse fly habitat suitability across Kenya. We applied four machine learning SDMs and an ensemble one that combined the four algorithms to predict the habitat suitability of *Glossina* spp. and *G. pallidipes* at a landscape scale. These provide updated predicted tsetse fly habitat suitability distribution to guide surveillance operations and support progressive control in Kenya. The four SDMs performed robustly with varied uncertainties in the predictions (Additional file 4: Fig. S3). Despite these notable variations, the SDMs (i.e., RF, SVM, MaxEnt, and GLM) indicated agreement levels at known tsetse fly locations. The RF model demonstrated high predictive power, capturing known tsetse fly hotspots, but indicated levels of over- and under-predictions across the country. These variations and uncertainties in the predicted outcomes are anticipated since models are based on different prediction algorithms [51].

The predictions were consistent with known tsetse fly occurrence locations across the country [18]. This indicated the robustness of our ensemble modeling approach in identifying areas likely to be infested with tsetse flies. Therefore, the covariates used in our modeling could explain *Glossina* spp. and *G. pallidipes* habitat distribution in the country. *Glossina pallidipes* could be precisely located in the predicted suitable habitats, which are more specific than the generalized genus-level predictions [52]. This indicates that the tsetse fly habitat distribution range can be better predicted and isolated at the species level to inform accurate mapping and location-specific control measures. Considering the shift in climate regimes, the tsetse fly habitat distribution could be altered; we thus need species-level maps to capture the possible ecological variability demonstrated by a single species.

This study demonstrated that temperature-related variables contributed fundamentally to predicting suitable habitats for the tsetse fly. The results indicated a negative association between tsetse fly habitat suitability and maximum temperature and elevation variables, where elevation beyond 400 m and temperature above 35 °C reduced tsetse fly habitat suitability scores. Our findings concur with Terblanche et al. [53], who demonstrated that tsetse fly activities are affected by increased temperatures. Furthermore, Mugenyi et al. [6] asserted that elevation had a negative linkage with the occurrence of tsetse flies. Besides, tsetse fly suitability is restricted beyond cooler regions and is more pronounced in lower elevations experiencing moderately warmer temperatures across landscapes [53]. This agrees with our findings that the highly suitable tsetse fly habitats are found in relatively lowlands of the country compared with the highlands, where suitability was predicted as moderate-to-low.

Soil moisture and vegetation cover are particularly critical in the breeding and sheltering of tsetse flies at a landscape scale [54]. The soil moisture-related variables demonstrated a positive correlation with tsetse fly distribution, with occurrence influenced by soil moisture levels ranging from 5 to 25 mm (Fig. 5). It is known that soil moisture availability drives the ecological requirements for tsetse flies, as it regulates temperatures and reduces the chances of desiccation, favoring their development [11]. In addition, the presence of vegetation enhances the ability of soils to retain water and provides a cooling effect that controls daytime temperatures [11]. In this study, suitability increased with increased vegetation cover, as areas with NDVI values greater than 0.6 showed high tsetse fly suitability (Fig. 5). This observation suggests that tsetse flies prefer shaded places after feeding, with limited feeding during hot afternoons when temperatures are high [55]. Nevertheless, subsurface soil moisture negatively affected the tsetse fly predictions across the study area. It was demonstrated that subsurface soil moisture exceeding 40 mm reduced tsetse fly habitat suitability substantially, implying that water-saturated soils could affect tsetse fly development. This finding aligns with Gachoki et al. [18], who reported that *Glossina* spp. thrives in places with moist soils to sustain pupae maturity, unlike waterlogged and dry soils [18].

In general, studies have demonstrated a positive correlation between tsetse fly presence and protected areas [6, 56]. Our study findings also showed highly suitable tsetse fly habitats around protected areas such as community forest reserves and game reserves across the country (Fig. 6). Practically, protected areas are characterized by different wildlife species of preference (such as buffaloes and warthogs) [52] and dense vegetation comprising woodlands, a preferred landcover type by *Glossina* spp., with limited human influence [18]. On the contrary, densely populated areas exhibit elevated levels of human activities that interfere with the preferred tsetse fly habitats, discouraging their establishment. In this study, the human population density variable showed a negative association with the predicted occurrence of tsetse flies. Specifically, tsetse fly habitat suitability decreased as human density increased to 1,000 people km^−2^. Similarly, other potential tsetse fly hosts, such as cattle and goat, penalized the model negatively, reinforcing the apparent association between these hosts and human settlements, vector control measures, and presumably applications of insecticides and acaricides. For instance, cattle densities from 7,500 to 10,000 km^−2^ reduced tsetse fly habitat suitability. Portions of highly fragmented tsetse fly-suitable habitats observed across the landscape could be small microhabitat pockets of forested areas scattered in open agricultural fields that intertwine agrilivestock production systems. These are potential grounds for highly isolated and increased trypanosome infections that risk small-scale farmers nationwide.

Specifically, our predictions showed high tsetse fly suitability in Maasai Mara, Tsavo East/West, Ruma, Amboseli, Nguruman, Dandori, and Meru national parks and wildlife conservancies, among other places in Kenya. These locations need to be surveyed as tsetse fly potential risk areas or habitats for future colonization, as observed from the novel zones (green grids) demarcated in Fig. 6a, b. We know that there is increased transmission of trypanosomiasis among cattle near protected areas [10]. Moreover, Warrier et al. [57] reported that about 62% of farmers adjoining Maasai Mara National Park and communities near protected areas gain access during the dry seasons in Kenya. In summary, integrating the novelty of the highly localized and precise resolution models produced in this study with tsetse fly progressive control measures would reduce tsetse fly infestation and AAT across hotspot regions. Counties such as Narok, Kajiado, and Kitui, spanning arid and semi-arid areas in Kenya, would benefit from highly localized interventions informed by accurate predictions.

Despite the relatively fine resolution of the tsetse fly habitat suitability maps produced, our study faced shortcomings in the spatial resolution of predictor variables. Most of the freely available satellite-based ecological variables are at a coarse spatial resolution of 4.6–10 km, which could limit the mimicry of the variability of finer landscape and climate dynamics. Such coarse resolution predictors could be downscaled to much finer layers using the advanced machine learning Earth observation processing techniques. In addition, the study could have benefited from wildlife density distribution data for more information on the preferred wild host. Lastly, documented tsetse fly observations were not well distributed across the country, as most of the northern parts were not covered; hence, more data from such landscapes could offer information lacking in these areas to our predictions. However, our approach applied herein attempts to complement such gaps by estimating the relationships of similar conditions where tsetse flies occur. Ultimately, these improved and updated tsetse fly predictions of relatively fine spatial resolution present an opportunity to upscale control measures targeting the deployment of various technologies at a highly localized scale (i.e., 1 × 1 km) compared with the maps currently used at a relatively coarse resolution (about 5 × 5 km).

## Conclusions

Using an ensemble modeling approach, this study predicted the spatial habitat distribution of tsetse flies in Kenya. This helped complement the updated tsetse fly atlases in areas of low data availability. Protected areas, conservancies, and forest reserves were identified as the affected locations with high tsetse fly suitability levels. Fragmented tsetse fly habitats observed could be associated with the shrinking habitats generated by human activity and the remaining focal point of continuous AAT disease transmission. Our results suggest that human activities are highly shaping the distribution of tsetse flies. This presents an opportunity to eliminate tsetse flies through progressive location-specific interventions and surveillance operations guided by these predictive maps at a relatively finer scale. It is anticipated that continued climate change will influence tsetse fly habitats, and species-level habitat maps are necessary to update the existing knowledge. Consequently, to capture future habitat outlook, suitability models of *G. pallidipes* should integrate robust predictive approaches and climate-simulated scenarios to accommodate the resulting climatic variations. The novel tsetse fly zones identified need more surveillance efforts to test the model’s applicability in areas where data are missing.

## Supplementary Information

Additional file 1: Table S1. A summary of the various predictor variables, data sources, timeline, and resolution levels in predicting potential habitat distribution for *Glossina *spp. and *G. pallidipes* in Kenya. (NDVI = normalized difference vegetation index; LST = land surface temperature; TWI = topographic wetness index).

Additional file 2: Fig. S1. A collinearity matrix of the variables was used to predict tsetse flies habitat suitability after performing a variance inflation factor (VIF) analysis, which excluded variables with a value => 10. The darker shading of blue and red demonstrated a high correlation, whereas the lighter shades indicated the least correlation. In this case, min = minimum; max = maximum; med = median; LST = land surface temperature; NDVI = normalized difference vegetation index.

Additional file 3: Fig. S2. Variable contribution analysis of the 10 most important predictor variables in four modeling algorithms, i.e., generalized linear model (GLM), random forest (RF), support vector machines (SVM), and maximum entropy (MaxEnt) used in predicting potential habitat for *Glossina* spp. (a – d) and *G. pallidipes* (e – h) in Kenya. In this case, min = minimum; max = maximum; med = median; LST = land surface temperature; NDVI = normalized difference vegetation index; SSMOIST = surface soil moisture.

Additional file 4: Fig. S3. Tsetse flies spatial habitat suitability in Kenya was predicted using four species distribution modeling algorithms, namely generalized linear models (GLM), random forest (RF), support vector machines (SVM), and maximum entropy (MaxEnt). a – d: *Glossina* spp. and e – h: *G. pallidipes*. The white shades indicate a low potential, the yellow shades indicate a moderate potential, and the red shades indicate a high potential of habitat suitability distribution in Kenya.

## Data Availability

Data utilized in this manuscript, along with the scripts to generate the figures, are available at https://dmmg.icipe.org/dataportal/dataset/species-distribution-modelling-to-predict-tsetse-fly-glossina-spp-habitat-suitability-in-kenya.

## Abbreviations

AAT: African animal trypanosomosis
AIC: Akaike information criterion
AOA: Area of applicability
AUC: Area under the curve
GIS: Geographic information system
GLM: Generalized linear models
HAT: Human African trypanosomosis
*icipe*: International Centre of Insect Physiology and Ecology
KENTTEC: Kenya Tsetse and Trypanosomiasis Eradication Council
LST: Land surface temperature
MaxEnt: Maximum entropy
NDVI: Normalized difference vegetation index
QGIS: Quantum Geographic Information System
RF: Random forests
SDM: Species distribution models
SSA: Sub-Saharan Africa
SVM: Support vector machines
TSS: True skill statistic
VIF: Variance inflation factor
