# Supplementary material for: Species distribution modeling to predict tsetse fly (Glossina spp.) habitat suitability in Kenya
Source: Parasit Vectors. 2025 Sep 24;18:378. doi: 10.1186/s13071-025-06938-1 (PMC12462162; doi:10.1186/s13071-025-06938-1)
Supplement: Supplementary file 1 — Additional file 1: Table S1. A summary of the various predictor variables, data sources, timeline, and resolution levels in predicting potential habitat distribution for Glossina spp. and G. pallidipes in Kenya. (NDVI = normalized difference vegetation index; LST = land surface temperature; TWI = topographic wetness index). [file 13071_2025_6938_MOESM1_ESM.docx]

**Additional file 1: Table S1**

| Variable | Hypothesis/Rationale | Data source | Timeline | Resolution | Unit |
| --- | --- | --- | --- | --- | --- |
| NDVI^b^ | NDVI is a greenness index used to infer vegetation performance. Vegetation provides shelter and maintains moist conditions for tsetse breeding. We hypothesized that places with high NDVI values would positively influence tsetse flies distribution. | Moderate Resolution Imaging Spectroradiometer (MODIS): <https://lpdaac.usgs.gov/products/mod13a2v061/> | 2017 – 2020 | 1 km | NU^a^ |
| LST^b^ | The life cycle of tsetse flies is influenced by temperature, where extreme conditions (i.e., either low or high) affect the growth rate and metabolic activities [11]. We hypothesized that areas with high and low temperatures will reduce the probability of tsetse occurrence. Temperatures below 10^0^C and above 36^0^C would affect the distribution of tsetse flies [53]. | National Aeronautics and Space Administration (NASA) land processes distributed active archive (LP DAAC) at the United States Geological Survey (USGS) Earth Resources Observation and Science (EROS) center:  <https://lpdaac.usgs.gov/resources/data-action/modis-land-surface-temperature-version-comparisons-appeears/> | 2017 – 2020 | 1 km | Kelvin |
| Air temperature^b^ | The life cycle of tsetse flies is influenced by temperature, where extreme conditions (i.e., either low or high) affect the growth rate and metabolic activities [11]. We hypothesized that areas with high and low temperatures will reduce the probability of tsetse occurrence. Temperatures below 10^0^C and above 36^0^C would affect the distribution of tsetse flies [53]. | Climate data store: <https://cds.climate.copernicus.eu/cdsapp#!/dataset/reanalysis-era5-land?tab=form> | 2017 – 2020 | 10 km | Kelvin |
| Soil temperature^b^ | Soil temperatures have an impact on the pupae development, where high and low temperatures would slow the rate. We hypothesized that areas with high and low soil temperatures will reduce the probability of tsetse occurrence. Temperatures below 10^0^C and above 36^0^C would affect the distribution of tsetse flies [53]. | Climate data store: <https://cds.climate.copernicus.eu/cdsapp#!/dataset/reanalysis-era5-land?tab=form> | 2017 – 2020 | 10 km | Kelvin |
| Precipitation^c^ | Precipitation provides ideal conditions for tsetse development and survival. It alters the humidity, vegetation growth, soil moisture, and overall temperatures, which are key factors in the occurrence of tsetse flies. We hypothesized that areas that receive moderate precipitation would be associated with tsetse flies distribution while areas that experience excessive precipitation would be negatively associated. | Climate data store: <https://cds.climate.copernicus.eu/cdsapp#!/dataset/reanalysis-era5-land?tab=form> | 2017 – 2020 | 10 km | Meter |
| Surface soil moisture^b^ | Moist conditions provide optimal conditions for pupae development, with very dry soils likely to suffocate pupae, while overly wet soils cause pupae drowning. We hypothesized that areas with extreme conditions would reduce the probability of tsetse flies occurrence. | NASA Goddard Space Flight Center: <https://developers.google.com/earth-engine/datasets/catalog/NASA_USDA_HSL_SMAP10KM> | 2017 – 2020 | 10 km | Millimeter |
| Sub-surface soil moisture^b^ | Moist conditions provide optimal conditions for pupae development, with very dry soils likely to suffocate pupae, while overly wet soils cause pupae drowning. We hypothesized that areas with extreme conditions would reduce the probability of tsetse flies occurrence. | NASA Goddard Space Flight Center: <https://developers.google.com/earth-engine/datasets/catalog/NASA_USDA_HSL_SMAP10KM> | 2017 – 2020 | 10 km | Millimeter |
| Skin temperature^b^ | Tsetse flies life cycle is influenced by temperature, where extreme conditions (i.e. either low or high) affect the growth rate and metabolic activities. We hypothesized that areas with high and low temperatures will reduce the probability of tsetse occurrence. Temperatures below 10^0^C and above 36^0^C would affect the distribution of tsetse flies [53]. | Climate data store: <https://cds.climate.copernicus.eu/cdsapp#!/dataset/reanalysis-era5-land?tab=form> | 2017 – 2020 | 10 km | Kelvin |
| Surface run-off**^c^** | Increased surface run-off increases the potential of washing away buried pupae. We hypothesized that areas with potent soil water saturation with high chances of increased surface run-off would be negatively correlated with tsetse flies distribution, especially during wet seasons. | Climate data store: <https://cds.climate.copernicus.eu/cdsapp#!/dataset/reanalysis-era5-land?tab=form> | 2017 – 2020 | 10 km | Meter |
| Sand content | Tsetse flies burrow into the soil to deposit pupae for sustained development. Loose sand soil is less likely to sustain enough moisture content than moderately compact soil. We hypothesized that areas with loose sand soil would be negatively associated with tsetse flies presence compared to well-drained and moderately compact soils. | Innovative Solutions for Decision Agriculture (iSDA): <https://developers.google.com/earth-engine/datasets/catalog/ISDASOIL_Africa_v1_sand_content> | 2017 | 30 m | Percentage |
| Elevation | Elevation affects the vegetation types, soil moisture, and temperatures. The warm slopes provide more suitable conditions for the tsetse survival than the cooler slopes. We hypothesized that warmer areas would be positively associated with tsetse flies presence. | LP DAAC USGS:  <https://lpdaac.usgs.gov/products/srtmgl1v003/> | 2000 | 30 m | Meter |
| Human population density | Human activities are linked to distracting and destroying tsetse flies habitats, limiting their development. We hypothesized that areas densely populated would be negatively correlated with the presence of tsetse flies. | World pop: <https://www.worldpop.org/> | 2020 | 1 km | NU^a^ |
| Cattle; sheep; goat density | Tsetse flies feed on livestock animals for their survival. Hence, the presence of these hosts would increase the probability of tsetse flies presence. | Food and Agriculture Organization of the United Nations (FAO): <https://www.fao.org/livestock-systems/global-distributions/en/> | 2015 | 10 km | Number km^-2^ |
| Protected areas (Euclidean distance) | Protected areas provide breeding grounds and habitats for wildlife species, with the preferred hosts for tsetse flies to feed. We hypothesized that areas close to protected areas would correlate positively with tsetse flies distribution. | Intergovernmental Authority for Development Climate Prediction and Applications Centre (ICPAC) geoportal: <https://geoportal.icpac.net/layers/geonode:ken_protected_areas> | Not applicable | 1km | Meter |
| TWI | TWI provides a landscape structural analysis identifying and quantifying sinks that could potentially hold water for causing flooding. We hypothesized that areas with a high probability of holding water would negatively affect the distribution of tsetse flies. Pupae development is adversely impacted by waterlogged soils that limit their development [18]. | Derivative of elevation data from USGS. | Not applicable | 30 m | Meter |
| ^a^ No Unit; ^b^ minimum, maximum and median; ^c^ sum | | | | | |
