# Supplementary figures and images for: Species distribution modeling to predict tsetse fly (Glossina spp.) habitat suitability in Kenya
Source: Parasit Vectors. 2025 Sep 24;18:378. doi: 10.1186/s13071-025-06938-1 (PMC12462162; doi:10.1186/s13071-025-06938-1)

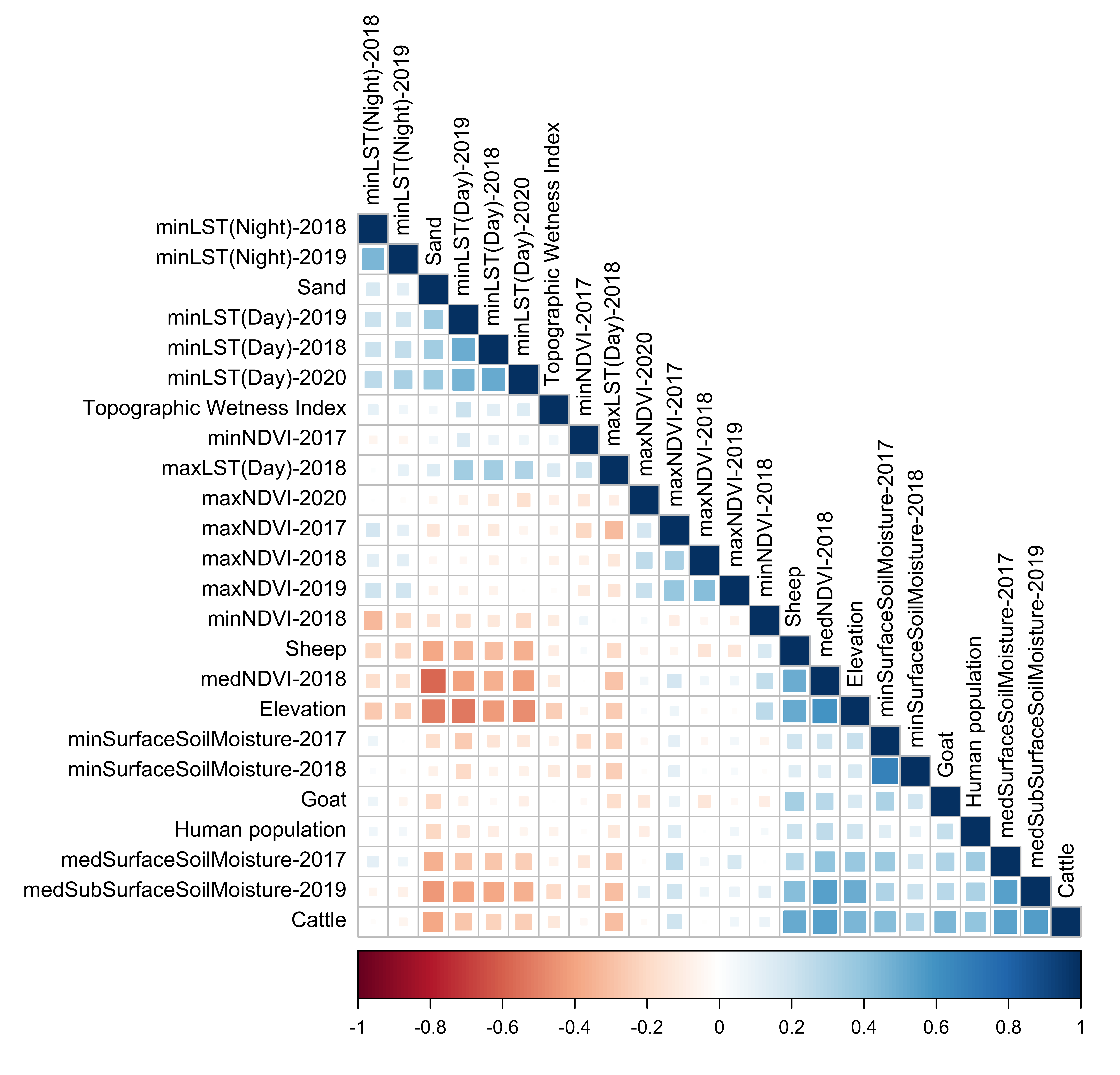

Supplement: Supplementary file 2 — Additional file 2: Fig. S1. A collinearity matrix of the variables was used to predict tsetse flies habitat suitability after performing a variance inflation factor (VIF) analysis, which excluded variables with a value => 10. The darker shading of blue and red demonstrated a high correlation, whereas the lighter shades indicated the least correlation. In this case, min = minimum; max = maximum; med = median; LST = land surface temperature; NDVI = normalized difference vegetation index. [file 13071_2025_6938_MOESM2_ESM.tiff]

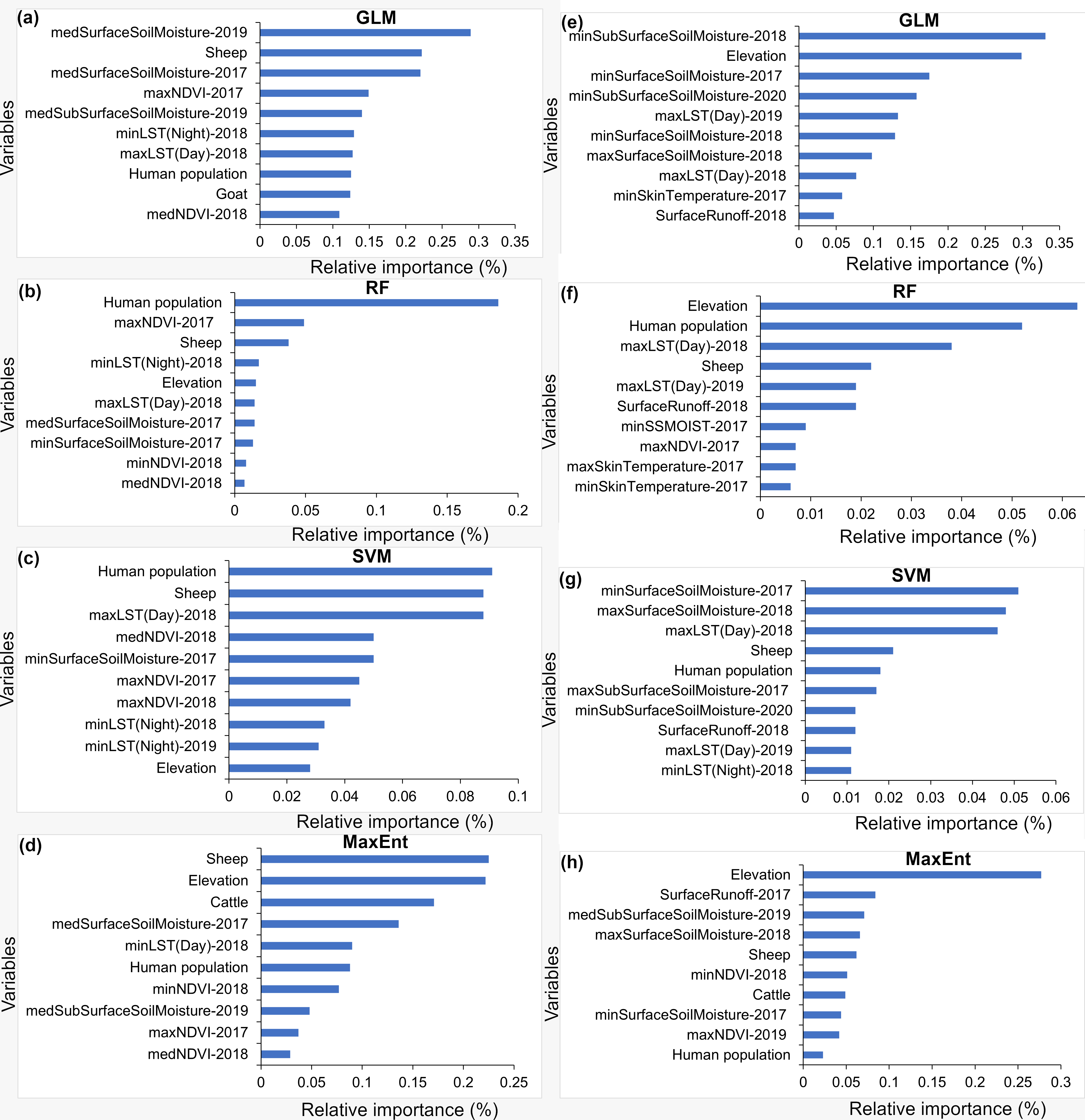

Supplement: Supplementary file 3 — Additional file 3: Fig. S2. Variable contribution analysis of the 10 most important predictor variables in four modeling algorithms, i.e., generalized linear model (GLM), random forest (RF), support vector machines (SVM), and maximum entropy (MaxEnt) used in predicting potential habitat for Glossina spp. (a – d) and G. pallidipes (e – h) in Kenya. In this case, min = minimum; max = maximum; med = median; LST = land surface temperature; NDVI = normalized difference vegetation index; SSMOIST = surface soil moisture. [file 13071_2025_6938_MOESM3_ESM.tiff]
